# Low-Loss Nanoscopic Spin-Wave Guiding in Continuous Yttrium Iron Garnet Films

**DOI:** 10.1021/acs.nanolett.2c01238

**Published:** 2022-06-21

**Authors:** Huajun Qin, Rasmus B. Holländer, Lukáš Flajšman, Sebastiaan van Dijken

**Affiliations:** † NanoSpin, Department of Applied Physics, Aalto University School of Science, P. O. Box 15100, FI-00076 Aalto, Finland; ‡ School of Physics and Technology, Wuhan University, Wuhan 430072, China; § Wuhan Institute of Quantum Technology, Wuhan 430206, China

**Keywords:** magnonics, spin-wave transport, magnonic waveguide, yttrium iron garnet

## Abstract

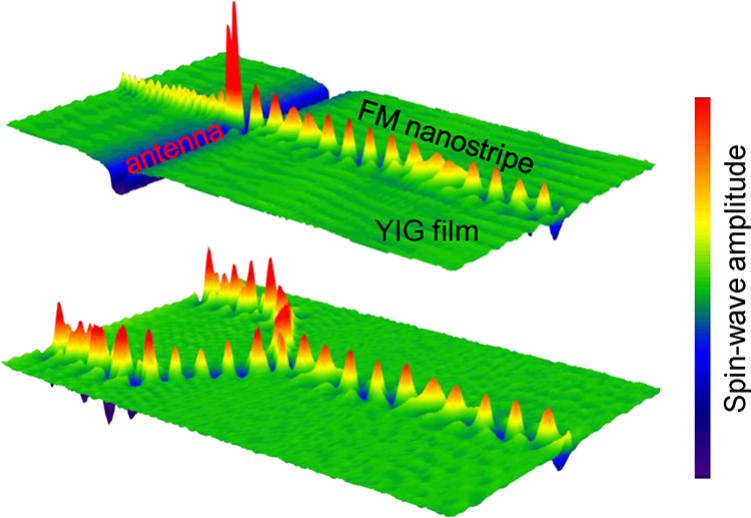

Long-distance transport and control of spin waves through nanochannels is essential for integrated magnonic technology. Current strategies relying on the patterning of single-layer nano-waveguides suffer from a decline of the spin-wave decay length upon downscaling or require large magnetic bias field. Here, we introduce a new waveguiding structure based on low-damping continuous yttrium iron garnet (YIG) films. Rather than patterning the YIG film, we define nanoscopic spin-wave transporting channels within YIG by dipolar coupling to ferromagnetic metal nanostripes. The hybrid material structure offers long-distance transport of spin waves with a decay length of ∼20 μm in 160 nm wide waveguides over a broad frequency range at small bias field. We further evidence that spin waves can be redirected easily by stray-field-induced bends in continuous YIG films. The combination of low-loss spin-wave guiding and straightforward nanofabrication highlights a new approach toward the implementation of magnonic integrated circuits for spin-wave computing.

Magnonics based on the transfer of angular momentum in the form of spin waves provides a promising technology for wave-based information processing^1−5^ and neuromorphic computing.^6,7^ Several signal processing devices utilizing the interference or nonlinear dynamics of spin waves have already been realized.^8−13^ Moreover, because spin-wave propagation does not produce ohmic losses, it can be more energy efficient than charge-carrier transport in complementary metal-oxide-semiconductor (CMOS) circuits. A key challenge in realizing a viable spin-wave technology is the connection of multiple computational units into integrated magnonic circuits, which requires a scalable materials platform for low-loss spin-wave transport. To be competitive, the transporting solution should enable spin-wave transmission through nanoscopic channels and allow signal redirection, interference, and manipulation.

The decay length (*l*_d_) of propagating spin waves in magnetic materials is proportional to the product of the group velocity (*v*_g_) and the spin-wave relaxation time (τ). Because τ is inversely proportional to the Gilbert damping parameter (α) and the frequency (*f*), *l*_d_ ∝ *v*_g_/*αf*. Ferromagnetic metals can provide high group velocity, but their damping parameter is often large, with the exception of specific 3*d* transition metal alloys^14,15^ and Heusler compounds.^16^ Waveguides made of ferromagnetic metals have been studied extensively,^17−22^ and because they are patterned easily, various waveguiding structures have been proposed.^23−25^ Moreover, metallic waveguides allow for the use of electric currents to guide^8^ or excite^26,27^ spin waves. Insulating yttrium iron garnet (YIG), on the other hand, offers ultralow Gilbert damping. Patterning of YIG films into nanoscopic waveguides and directional couplers has been demonstrated recently,^28−31^ paving the way toward low-loss magnonic devices. However, the patterning of nanoscale YIG structures remains challenging because its damping parameter easily deteriorates by defect formation during ion milling. Apart from extrinsic effects caused by nanopatterning, there are also fundamental challenges in transporting spin waves along laterally confined nanostructures. For waveguides wherein the magnetization aligns along the transporting direction, the decay length drops quickly below a width of 1 μm because of a declining group velocity,^28^ while waveguides with transverse magnetization require an ever-increasing magnetic bias field upon downscaling. For technologically relevant waveguides with a width below 200 nm, the spin-wave decay length is typically limited to 1–3 μm for ferromagnetic metals and YIG.^28,32^ The largest decay length in ultranarrow waveguides is 8 μm, which was measured recently on YIG in a narrow frequency range and at a large bias field of 270 mT.^31^ Magnetic domain walls, which have been proposed as nanochannels for spin-wave guiding, also restrict the propagation distance of spin waves to a few micrometers.^33−36^

Here, we experimentally demonstrate a new low-loss magnonic waveguiding structure. In our approach, spin waves propagate in a continuous YIG film along nanoscopic channels that are defined by dipolar coupling to a ferromagnetic metal nanostripe patterned on top. By taking advantage of ultralow damping in YIG and avoiding any nanopatterning of this oxide material, we demonstrate a spin-wave decay length of ∼20 μm in 160–400 nm wide waveguiding structures at a modest bias field of 25 mT. Additionally, our hybrid materials platform enables low-loss spin-wave transport through curved nanowaveguides.

Figure 1a illustrates the hybrid waveguiding structure and the experimental configuration for the imaging of spin-wave transport. The waveguide consists of a continuous YIG film with a CoFeB nanostripe patterned on top. To restrict the interaction between YIG and CoFeB solely to dipolar coupling fields, we use a 6 nm thick TaO_*x*_ spacer to separate the two magnetic materials. The interlayer coupling strength is controlled by an external magnetic field, which is applied along the *y* axis, perpendicular to the CoFeB nanostripe (Figure 1b). In this configuration, a field of ∼1 mT saturates the magnetization of the YIG film perpendicular to the waveguide, thus establishing spin-wave transport in the Damon–Eshbach geometry. Moreover, coherent rotation of the CoFeB magnetization away from the stripe axis in an external field tunes the spin-wave properties through a controlled variation of the dipolar coupling strength. In our experiments, the YIG film is 66 nm thick and its Gilbert damping parameter is 5 × 10^–4^ (Figure S1 in the Supporting Information (SI)). The thickness of the CoFeB nanostripe is 24 nm, and we vary the stripe width from 400 nm down to 160 nm. Spin waves are excited by a 1 μm wide microwave antenna, and their propagation is imaged using super-Nyquist sampling magneto-optical Kerr effect (SNS-MOKE) microscopy (see Methods in the SI).

**Figure 1:**
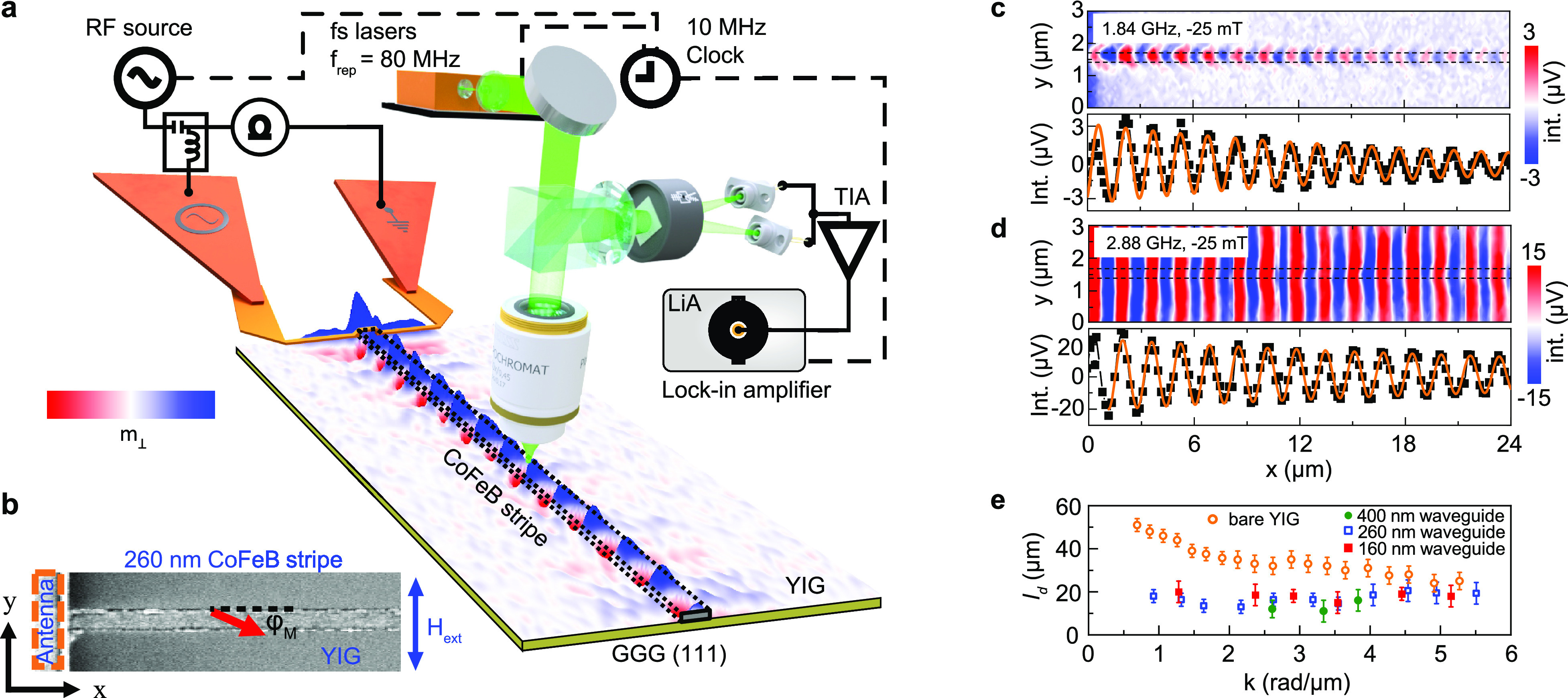
Long-distance spin-wave transport in magnonic nano-waveguides. (a) Schematic of the experiment. The waveguide consists of a continuous YIG film with a CoFeB nanostripe patterned on top. Dipolar coupling between the two magnetic materials induces a one-dimensional nanochannel with reduced effective magnetic field in the YIG film, along which spin waves propagate with low damping below the FMR frequency of the uncovered YIG film. Propagating spin waves are excited by a microwave antenna and imaged by SNS-MOKE microscopy. An external magnetic field (*H*_ext_) is applied perpendicular to the waveguide. (b) Scanning electron microscopy image of a waveguiding structure. The red arrow indicates the magnetization angle, φ_M_, in the CoFeB nanostripe with respect to the *x* axis. (c and d) SNS-MOKE microscopy maps and line profiles of propagating spin waves in a 260 nm wide waveguide at 1.84 and 2.88 GHz. The external magnetic field is −25 mT. The dashed lines indicate the CoFeB stripe. The orange lines depict fits to the experimental data. (e) Wave-vector dependence of the spin-wave decay length in hybrid waveguides of different width and in the uncovered YIG film. The bias field is −25 mT.

An example of spin-wave transport along a 260 nm wide waveguiding structure is shown in Figure 1c. At an excitation frequency of 1.84 GHz and −25 mT magnetic field, spin waves only propagate along a narrow channel that is defined by the CoFeB nanostripe. Localization of spin-wave transport in the YIG film breaks down at higher frequency (Figure 1d), where spin waves are excited across the entire length of the microwave antenna. When guided along a narrow channel, spin waves propagate over a long distance that is only reduced by a factor of 1–2.5 compared to nonlocalized transport in the uncovered YIG film (Figure 1e, the variation of *l*_d_ with frequency is depicted in Figure S2 of the SI). Fitting the spin-wave amplitude in the waveguide to *C* exp(− |*x*|/*l*_d_) sin(2*πx*/λ + ϕ), we extract spin-wave decay length *l*_d_ ≈ 20 μm, which is larger than the decay lengths measured on patterned single-layer nanoscopic waveguides.^28,31,32^ Moreover, we find that the propagation of spin waves does not vary strongly with wave vector, frequency, or channel width, as similar transport characteristics are measured on 400 nm wide and 160 nm wide waveguides (see Figure 1e; SNS-MOKE microscopy measurements underlying the data are shown in Figures S3 and S4 of the SI). The nearly constant spin-wave decay length in the waveguide is explained by the linear dispersion of spin waves in the CoFeB/YIG bilayer for this range of wave vectors, as we will discuss next.

To analyze spin-wave transport in the waveguides, we derive the dispersion relation from SNS-MOKE measurements. Figure 2a shows the experimental data together with results from micromagnetic simulations and theoretical calculations (see Methods in the SI) for −25 mT external magnetic field. Because dynamic dipolar coupling between the YIG film and CoFeB stripe depends on the chirality of propagating spin waves,^13,37−40^ transport along the waveguide is nonreciprocal. Localization of spin-wave transmission is enabled by a frequency downshift of the dispersion relation in the CoFeB/YIG bilayer region compared to that of the uncovered YIG film due to a local lowering of the effective magnetic field. Profiles of the effective field in the YIG film and CoFeB stripe are depicted in Figure 2b. For an external field of −25 mT, the magnetization in the CoFeB stripe is rotated by φ_M_ = 14° with respect to the *x* axis (Figure 2c). The stray field that this rotation produces reduces the effective field at the center of the waveguiding channel in the YIG film to just 3 mT. Spin waves propagate exclusively along this magnetically induced nanochannel up to the FMR frequency of the uncovered YIG film (2.25 GHz in Figure 2a). While any ferromagnetic stripe on top of YIG would downshift the spin-wave dispersion relation, the effect is particularly strong when its saturation magnetization is large. To ensure waveguiding over a broad frequency range, we selected CoFeB in our experiments.

**Figure 2:**
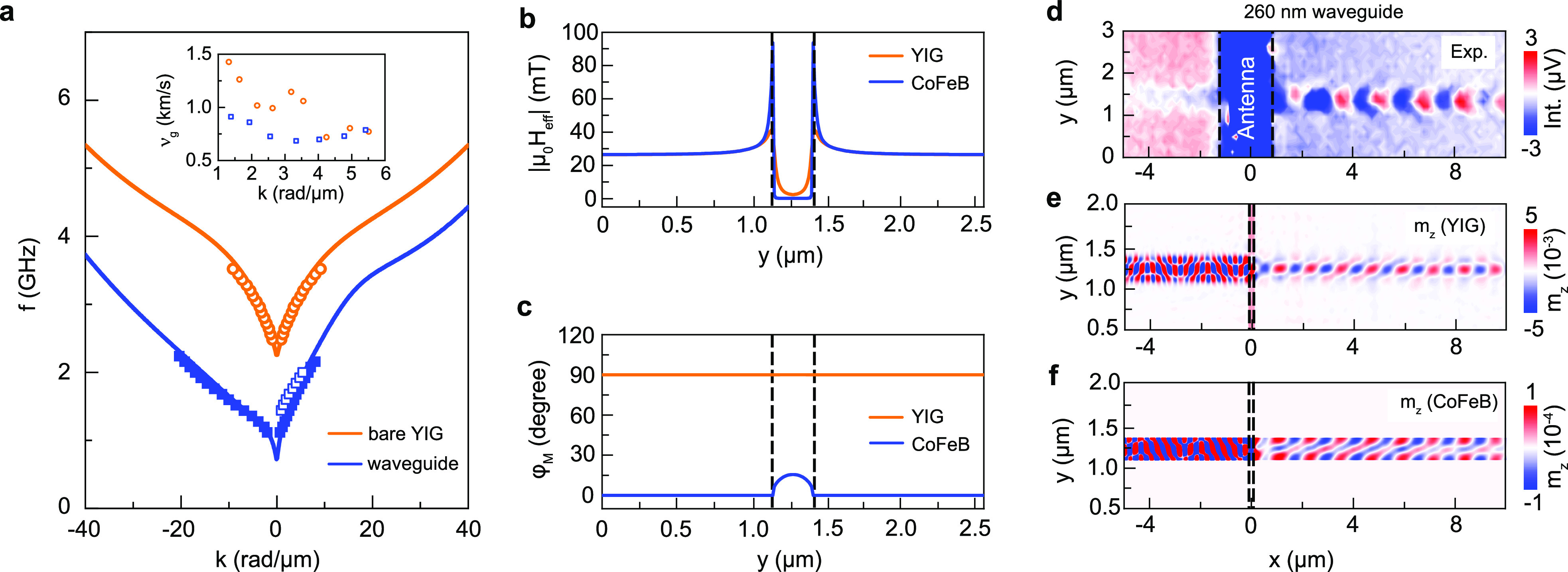
Spin-wave dispersion and confinement. (a) Measured (empty symbols), calculated (lines), and simulated (solid squares) spin-wave dispersion relations for a 66 nm thick bare YIG film and a 260 nm wide waveguide. The spin-wave group velocity shown in the inset is derived from the experimental data. (b and c) Simulated effective field and magnetization angle in the YIG film and a 260 nm wide CoFeB stripe. The magnetization angle is defined with respect to the *x* axis. The vertical dashed lines indicate the position of the CoFeB nanostripe. (d–f) Measured and simulated spin-wave maps for a 260 nm wide waveguide at 1.84 GHz. The micromagnetic simulations in panels e and f depict the spin-wave mode in YIG and CoFeB, respectively. All data are obtained for a magnetic field of −25 mT. Similar data sets for 160 nm wide and 400 nm wide waveguiding structures are shown in Figures S7 and S8 of the SI.

The spin-wave group velocity in the CoFeB/YIG waveguide is approximately constant in the measurement range, which contrasts with the group velocity in the uncovered YIG film (inset in Figure 2a). Considering *l*_d_ ∝ *v*_g_/*αf*, the following picture emerges; the group velocity of the CoFeB/YIG waveguiding structure is smaller than that of the uncovered YIG film at small wave vector. Together with stronger damping in CoFeB/YIG, this causes spin waves with *k* = 1 rad/μm to decay ∼2.5 times faster in the nanoscopic waveguide (Figure 1e). From the experimental data (decay length, group velocity, and frequency), we estimate α_WG_ ≈ 2.5α_YIG_. With increasing wave vector, the group velocitity in YIG drops while that of the CoFeB/YIG waveguide remains approximately constant. The group velocity of the waveguide thus compensates for its stronger damping, resulting in similar spin-wave decay lengths at *k* = 5 rad/μm (Figure 1e). This comparison illustrates that the propagation of spin waves along the hybrid waveguide can be optimized through engineering of the bilayer dispersion relation. Interestingly, micromagnetic simulations indicate that the decay length in the waveguiding structure does not depend on the damping parameter of the ferromagnetic nanostripe (Figure S5 in the SI). In contrast, variation of the YIG damping parameter sensitively tunes the spin-wave propagation distance along the magnetically induced nanochannel (Figure S6 in the SI). These results demonstrate that long-distance transport of confined spin waves is determined fully by the low-damping parameter of the continuous YIG film and that the material of the ferromagnetic nanostripe can be chosen freely.

Panels d–f of Figure 2 show the measured spin-wave profile at 1.84 GHz and corresponding layer-resolved simulations for the YIG film and 260 nm wide CoFeB nanostripe. Similar data sets for 160 nm wide and 400 nm wide waveguiding structures are summarized in Figures S7 and S8 of the SI. The data confirm nonreciprocal spin-wave transport as the wavelength is much shorter for −*x* than +*x*. The short-wavelength mode is not seen in the experiments because of the limited excitation range of the microwave antenna and the imaging resolution of SNS-MOKE microscopy. The propagating spin waves in YIG and CoFeB are out-of-phase, and the wave vector along the *y* axis is quantized in both layers. From the simulations, we conclude that spin-wave transport in the continuous YIG film is highly localized. While the wave profile along the *y* axis in YIG is a bit broader than the width of the CoFeB nanostripe, the broadening is limited to about 15%.

The dispersion of spin waves in the hybrid waveguiding structure varies with the strength of interlayer dipolar coupling, which depends sensitively on the direction of magnetization in the CoFeB nanostripe. Consequently, various spin-wave properties change when the external magnetic field increases. To quantify this effect, we measured the spin-wave dispersion (Figure 3a) and extracted the group velocity (Figure 3b) at different magnetic fields. The dispersion curve of the CoFeB/YIG waveguide and the uncovered YIG film at −75 mT are compared in Figure 3c. The data demonstrate that an increase of the external field enhances the frequency of spin waves in the waveguide, but that their group velocity remains approximately constant. The field-induced shift of the spin-wave dispersion relation is smaller for the waveguiding structure than for the YIG film. This difference is explained by coherent magnetization rotation in CoFeB, which enlarges the demagnetization field in CoFeB and the stray field in YIG, thereby partially compensating for the increasing external field (Figure 3d,e). The growing frequency gap between the two dispersion curves at large external field facilitates localization of short-wavelength spin waves up to high frequency (Figure 3f). For instance, localized spin waves with wavelengths down to 130 nm do propagate along the CoFeB/YIG waveguiding structure at −75 mT. Another notable field effect is the growing asymmetry of the spin-wave dispersion relation (see Figures 2a and 3c). The frequency nonreciprocity of the waveguiding structure increases with external magnetic field, as illustrated in Figure 3g for a wave vector of 7 rad/μm.

**Figure 3:**
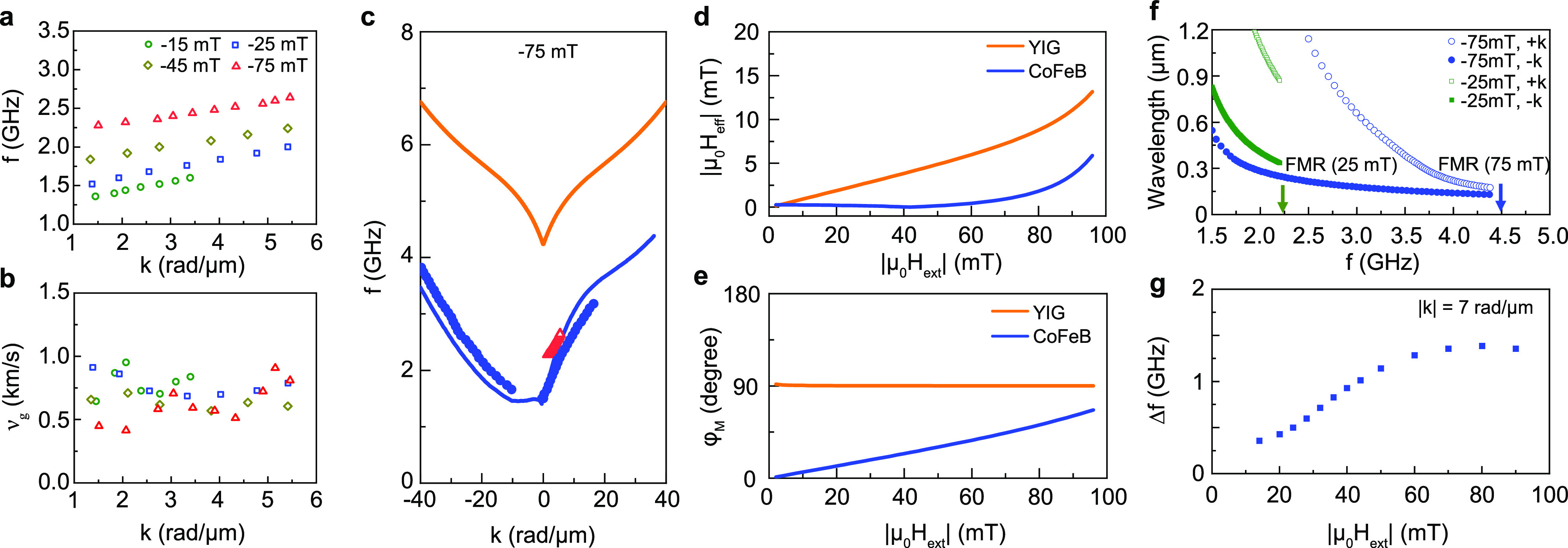
Magnetic field tuning of spin-wave properties. (a) Measured spin-wave dispersion relations for a 260 nm wide waveguide at different external magnetic fields. (b) Spin-wave group velocity derived from panel a. (c) Measured (empty symbols), calculated (lines), and simulated (solid squares) spin-wave dispersion relations for a 66 nm thick bare YIG film and a 260 nm wide waveguide at −75 mT magnetic field. (d and e) Magnetic field dependence of the effective field and magnetization angle in the YIG film underneath the CoFeB stripe and in the CoFeB stripe. (f) Wavelength of spin waves propagating along a 260 nm wide waveguide at −25 and −75 mT. Arrows mark the FMR frequencies of the uncovered YIG film, i.e., the frequency above which spin-wave transport is no longer localized. (g) Frequency nonreciprocity of spin waves with |*k*| = 7 rad/μm as a function of external magnetic field.

Magnonic circuits require low-loss transmission of spin waves in curved waveguides. Our bilayer structure allows the redirection of spin-wave transport in a continuous YIG film. As an example, we image the propagation of spin waves through waveguiding structures wherein 260 nm wide and 160 nm wide CoFeB nanostripes bend by 17° (Figure 4). In the tilted part of the waveguides, the wavelength of spin waves converts either up or down, depending on the direction of the bend, because of changes in the effective magnetic field (Figure S9 in the SI). Transport through the curve, however, hardly affects the transmission loss, as illustrated by the signal intensity measured on waveguiding structures with and without bend (blue data points in Figure 4g). Curved waveguides are an important building block of spin-wave interference devices.^1−5^ In Figure 4c,f, we image wave transport in narrow Y-shaped waveguides. In this configuration, constructive interference would enhance the spin-wave intensity after the two legs combine into one. We, however, measure a smaller spin-wave intensity in Y-shaped structures than in single waveguides with a bend (compare orange and blue data points in Figure 4g), signifying nonconstructive spin-wave interference. This effect is explained by slightly different effective magnetic fields in the two bends of the Y-shaped waveguide (Figure S9 in the SI). Tuning of the spin-wave interference condition is possible through a variation of the field strength or orientation, or through an optimization of the bend angles.

**Figure 4:**
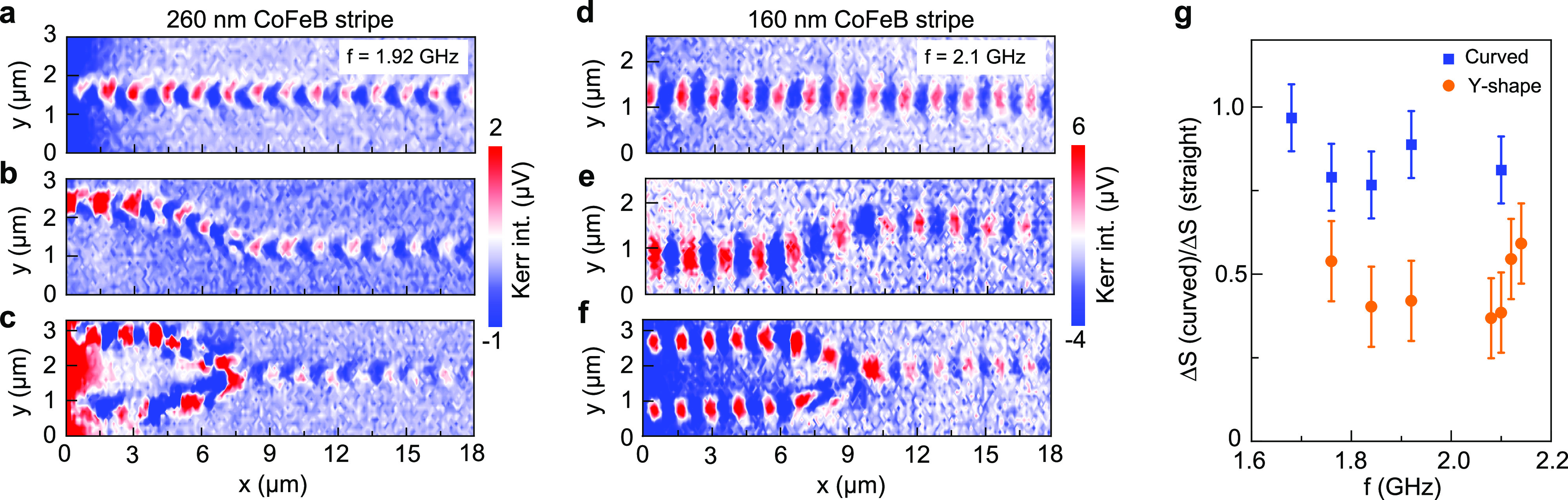
Spin-wave transport in curved nanowaveguides. (a–f) Comparison of spin-wave transport along straight, curved, and Y-shaped waveguides with a width of (a–c) 260 nm and (d–f) 160 nm. The angle of the curve is 17°, and the external magnetic field is −25 mT. (g) Ratio of the spin-wave amplitude after 9 μm transport along straight and curved waveguides (blue symbols). The orange data show the same ratio for Y-shaped waveguides.

Nanoscopic magnonic waveguides combining low-loss transport and straightforward nanofabrication are of great importance for the realization of spin-wave logic gates and unconventional magnonic computing devices.^41^ Fast nondissipative logic operations require the use of short-wavelength spin waves with high group velocity, magnonic nanostructures, and low-loss spin-wave transport. Bilayer waveguides made of a low-damping continuous YIG film and ferromagnetic metal nanostripes, as introduced here, offer many attractive properties. First, by avoiding patterning of the YIG film, spin-wave scattering on milling-induced defects is avoided. Second, the magnetization of the YIG film saturates perpendicular to the waveguiding structure in an ∼1 mT field. Because a small rotation of the CoFeB magnetization away from the nanostripe axis already localizes the transport of spin waves in the YIG film, only a modest bias field is needed. Third, the spin-wave decay length of the hybrid waveguiding structure is large compared to fully patterned nanoscopic waveguides. While dipolar coupling between CoFeB and YIG enhances the effective damping, this effect is compensated for, in part, by a different dependence of the group velocity on the wave vector. Our experiments demonstrate that the spin-wave decay length in a 160 nm waveguide is identical to the decay length of the low-damping continuous YIG film at *k* = 5 rad/μm (Figure 1e), which is an unprecedented result. Moreover, we find that the propagation distance of spin waves does depend on the damping parameter of the continuous YIG film, but not on the damping in the ferromagnetic nanostripe. Fourth, the hybrid waveguiding structure offers tunable frequency nonreciprocity, which could be used to isolate circuit components. Fifth, spin waves are transported efficiently through bends in the bilayer waveguide.

Besides these promising features, the hybrid waveguiding structure has two limitations: The spin-wave signal in the continuous YIG film is not fully confined to the width of the CoFeB nanostripe and localized spin-wave transport breaks down at the FMR frequency of the uncovered YIG film. Using experimental parameters in micromagnetic simulations, we estimate that the spin-wave signal in YIG is confined to about 400, 300, and 300 nm for the 400, 260, and 160 nm wide waveguides (Figure 2e and Figures S7 and S8 in the SI). We note, however, that these results are obtained for a 66 nm thick YIG film and a 24 nm thick CoFeB nanostripe. The confinement of spin waves improves considerably in thinner YIG films. For instance, micromagnetic simulations on a 160 nm wide waveguide comprising a 20 nm thick YIG film and a 15 nm thick CoFeB nanostripe indicate full confinement of propagating spin waves to a 160 nm wide channel in YIG (Figure S10 in the SI).

Spin-wave transport is only localized in YIG between the FMR frequencies of the bilayer structure and the uncovered YIG film. This restricts the wavelength of propagating spin waves. Yet, short wavelengths can still be attained. Our waveguiding structure supports the transport of confined spin waves with wavelengths down to 330 nm at a magnetic bias field of −25 mT and wavelengths down to 130 nm at −75 mT (Figure 3f), which is well into the dipolar exchange coupling regime. Waves with shorter wavelengths can be confined by increasing the magnetic field further or by modifying the bilayer dispersion curve through structural or material changes.

Finally, we note that hybrid low-loss waveguiding structures could also be realized by considering other magnetic coupling mechanisms. For the 66 nm thick YIG film in our experiments, dipolar coupling is most effective. Direct exchange at the CoFeB/YIG interface of structures without spacer, for instance, would only have a minor effect on the confinement of propagating spin waves (see Figure S11 in the SI). For thinner films, antiferromagnetic coupling effects that lower the effective field in YIG, such as RKKY coupling, may become attractive.

In summary, we demonstrated a low-loss hybrid materials platform for long-distance transport of spin waves along nanoscopic channels. Combining continuous YIG films with patterned ferromagnetic metal nanostripes offers straightforward fabrication and great flexibility in the design of desirable spin-wave properties, including long decay lengths, nonreciprocity, and efficient guiding through bends. Our work provides a new strategy for the implementation of low-loss magnonic devices and integrated circuits without YIG nanopatterning.

## Supporting Information Available

Methods and additional data, including ferromagnetic resonance measurements of the YIG film, spin-wave maps and profiles of different waveguiding structures, and micromagnetic simulations (PDF)
