# Supplementary material for: Low-Loss Nanoscopic Spin-Wave Guiding in Continuous Yttrium Iron Garnet Films
Source: Nano Lett. 2022 Jun 21;22(13):5294–300. doi: 10.1021/acs.nanolett.2c01238 (PMC9284617; doi:10.1021/acs.nanolett.2c01238)
Supplement: Supplementary file 1 — nl2c01238_si_001.pdf [file nl2c01238_si_001.pdf]

Low-loss nanoscopic spin-wave guiding in continuous YIG films

Huajun Qin,*,†,‡ Rasmus B. Holländer,† Lukáš Flajšman,† and Sebastiaan van Dijken*,†

†*NanoSpin, Department of Applied Physics, Aalto University School of Science, P.O. Box 15100, FI-00076 Aalto, Finland*

‡*School of Physics and Technology, Wuhan University, Wuhan 430072, China*

E-mail: huajun.qin@aalto.fi; sebastiaan.van.dijken@aalto.fi

Methods

Sample fabrication

We used PLD to grow 66-nm-thick YIG films on GGG(111) substrates. The GGG substrates were ultrasonically cleaned in acetone and isopropanol before loading into the deposition chamber. The substrates were degassed at 550°C for 15 min. After this, oxygen was inserted into the chamber and the temperature was raised to 800° at a rate of 5° per minute. YIG films were deposited from a stoichiometric target in an oxygen partial pressure of 0.13 mbar. We used an excimer laser with a pulse repetition rate of 2 Hz and a laser fluence of 1.8 J/cm^2. Following film growth, we first annealed the YIG films at 730° for 10 min in an oxygen environment of 13 mbar and then cooled them down to room temperature at a rate of $-3°$ per minute. The deposition process resulted in single-crystal YIG films, as confirmed by x-ray diffraction and transmission electron microscopy measurements. The

saturation magnetization of the YIG films was 181 kA/m and the Gilbert damping parameter was 5×10^{-4}, as extracted by ferromagnetic resonance spectroscopy (Figure S1). CoFeB nanostripes were fabricated on the YIG films by electron-beam lithography and lift-off in a bath of acetone. We used magnetron sputtering to grow 6 nm TaO_x and 24 nm $Co_{40}Fe_{40}B_{20}$ at room temperature. For spin-wave characterization, 1-μm-wide microwave antennas were patterned on top of the YIG films using electron-beam lithography and a lift-off. The antennas consisted of 3 nm Ta and 120 nm Au.

Super-Nyquist sampling magneto-optical Kerr effect microscopy

We used a home-built SNS-MOKE microscope to image spin-wave transport in the CoFeB/YIG waveguides and uncovered YIG films. In SNS-MOKE, the laser frequency comb downconverts the excited GHz magnetization dynamics to an intermediate frequency ϵ, allowing tuning of the excitation signal to any frequency $f_{\mathrm{exc}} = n \times f_{\mathrm{rep}} + \epsilon$. At non-zero ϵ and with the excitation synchronized to a laser repetition rate of 80 MHz, this downconversion occurs coherently, i.e., the phase of spin waves relative to the excitation signal is preserved by lock-in demodulation at ϵ.[1] Spin waves were excited at $P = 0$ dBm by a microwave antenna. The laser beam with a wavelength of 515 nm and a power of 2.6 mW was focused on the film surface with an effective spot diameter of 400 nm (500 nm) using a 100x (50x) microscope objective with a large numerical aperture (NA = 0.75 and 0.55). To record 2D spin-wave maps, the samples were moved with respect to the laser spot in the x and y directions using a piezoelectric stage. We used stabilization markers patterned near the CoFeB nanostripes to maintain the same relative position of the laser spot during the measurements.

Micromagnetic simulations

We performed micromagnetic simulations using open-source GPU-accelerated MuMax3 software.[2] The simulation structure consisted of a continuous YIG film, a spacer layer and a CoFeB stripe with respective thicknesses of 66 nm, 6 nm, and 24 nm. We discretized the

simulation area into $10 \times 10 \times 6$ nm^3 cells. The length of CoFeB stripe along the x axis was 40.96 μm and its width was 260 nm or 160 nm. One-dimensional periodic boundary conditions were applied along the y-axis to mimick the continuous YIG film. Spin-wave reflections from the edges along x were suppressed by the addition of two 5-μm-wide areas with strong magnetic damping ($\alpha = 0.5$). As input parameters, we used $M_s = 181$ kA/m, $A_{ex} = 3.1$ pJ/m and $\alpha = 0.001$ for YIG, $M_s = 1150$ kA/m, $A_{ex} = 16$ pJ/m and $\alpha = 0.005$ for CoFeB, and $M_s = 0$, $A_{ex} = 0$ pJ/m, and $\alpha = 10000$ for the spacer layer. The interlayer exchange interaction between the YIG film and the CoFeB nanostripe was set to zero. We used a sinusoidal ac magnetic field of 0.5 mT to locally excite spin waves. The excitation field was applied over a 1-μm-wide area through the whole film thickness at $x = 0$, as indicated in the figures.

Calculations of spin-wave dispersion relations

In order to calculate the spin-wave dispersion of the CoFeB/YIG waveguiding structure, we first performed static micromagnetic simulations to extract the effective field and magnetization direction in the YIG and CoFeB layers at the waveguide center. Together with the exchange stiffness, saturation magnetization, and thickness of each magnetic layer, we used these parameters as inputs in semi-analytical dispersion calculations based on the multilayer method developed by Arias.[3,4] In the calculations, we applied magnetostatic boundary conditions across the spacer layer and on the outer surfaces along the z-axis, and we used intralayer exchange boundary conditions within both magnetic layers. Also, the vanishing out-of-plane component of the local effective field and the interlayer exchange coupling A_{12} were set to zero. This parameter set resulted in a system of 12 equations and 12 variables. To find the dispersion relation, we numerically extracted the zero-crossings of the determinant. This was done by evaluating the determinant for frequency-wavevector combinations on a grid of 1001 frequencies between 0.25 GHz and 4.5 GHz and 1001 wavevectors between 10^{-3} μm^{-1} and 70 μm^{-1}. Because the determinant is a complex number, the zero-crossings could

be found as minima of the absolute value.

References

(1) Qin, H.; Holländer, R. B.; Flajšman, L.; Hermann, F.; Dreyer, R.; Woltersdorf, G.; van Dijken, S. Nanoscale magnonic Fabry-Pérot resonator for low-loss spin-wave manipulation. *Nat. Commun.* **2021**, *12*, 2293.

(2) Vansteenkiste, A.; Leliaert, J.; Dvornik, M.; Helsen, M.; Garcia-Sanchez, F.; Van Waeyenberge, B. The design and verification of MuMax3. *AIP Adv.* **2014**, *4*, 107133.

(3) Arias, R. E. Spin-wave modes of ferromagnetic films. *Phys. Rev. B* **2016**, *94*, 134408.

(4) Armijo, I. A.; Arias, R. E. Spin wave modes of multilayered ferromagnetic films. *Phys. Rev. B* **2019**, *99*, 014432.

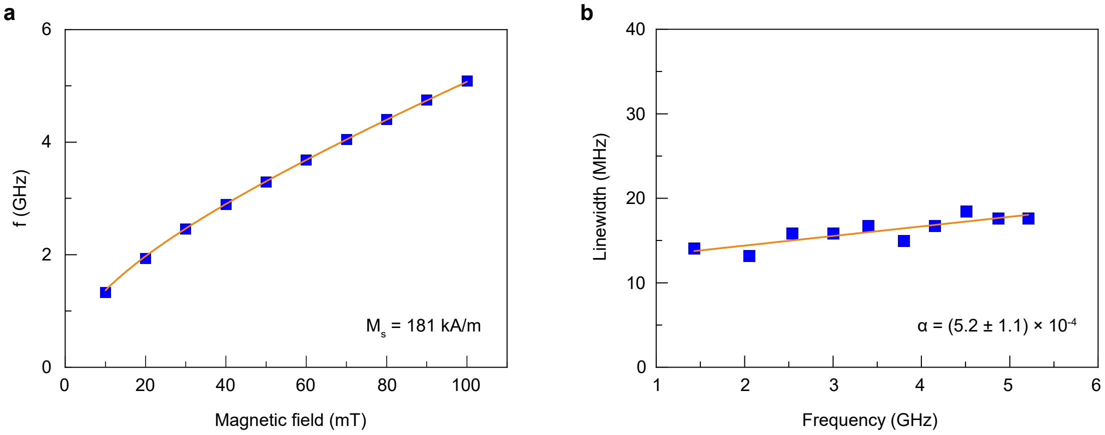

Figure S1. (a) Frequency of the YIG FMR mode as a function of external magnetic field measured by placing a 66-nm-thick YIG film face-down onto a coplanar waveguide. The Kittel-formula fit (orange line) of the experimental data gives a saturation magnetization $M_s = 181 \pm 5$ kA/m. (b) Linewidth of the YIG FMR mode as a function of frequency. A linear fit to the data using $\Delta f = 2\alpha f + \upsilon_g \Delta k$ yields a Gilbert damping constant $\alpha = (5.2 \pm 1.1) \times 10^{-4}$.

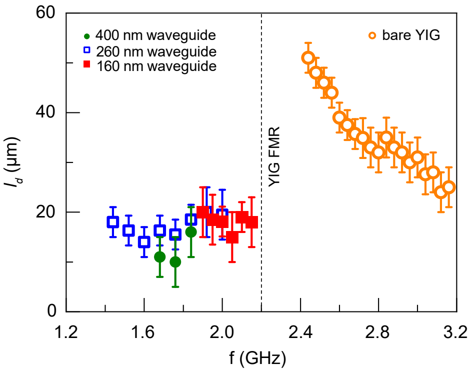

Figure S2. Frequency dependence of the spin-wave decay length in waveguides of different width and in the uncovered YIG film. The magnetic bias field is −25 mT. The dashed line marks the FMR frequency of the YIG film.

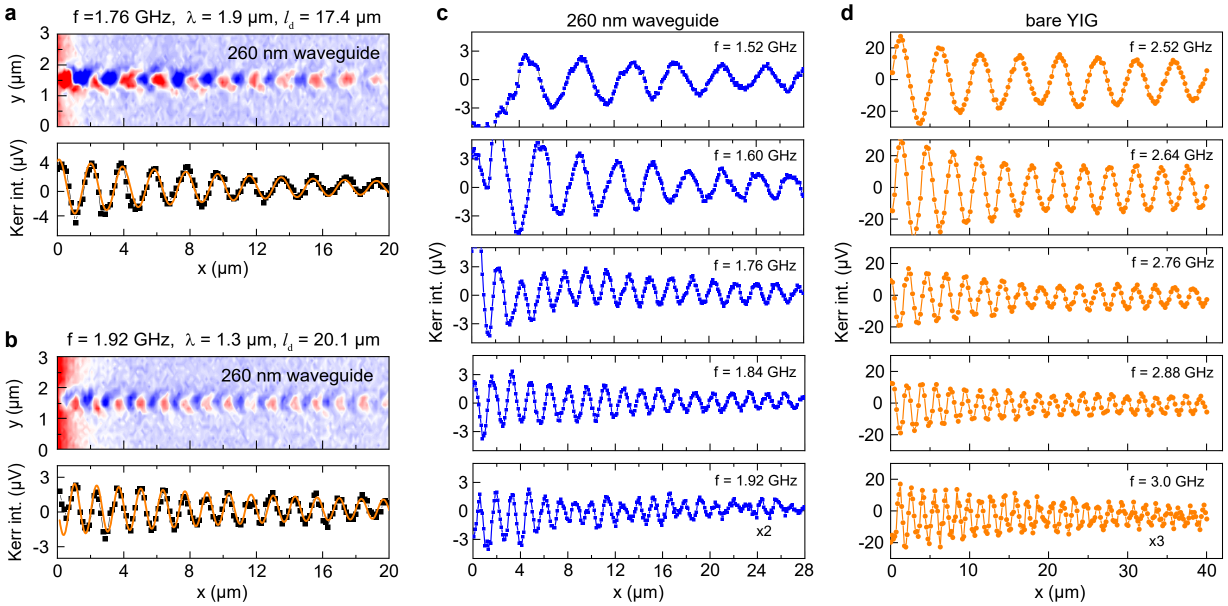

Figure S3. (a and b) SNS-MOKE microscopy maps and line profiles of propagating spin waves in a 260-nm-wide waveguide at 1.76 GHz and 1.84 GHz. The orange lines depict fits to the experimental data using $C \exp(-|x|/l_{\rm d}) \sin(2\pi x/\lambda + \phi)$. The fitted wavelength (λ) and spin-wave decay length ($l_{\rm d}$) are given above the spin-wave maps. (c) SNS-MOKE spin-wave profiles for a 260-nm-wide waveguide at different frequency. (d) SNS-MOKE spin-wave profiles for an uncovered YIG film at different frequency. All data are recorded at −25 mT external magnetic field.

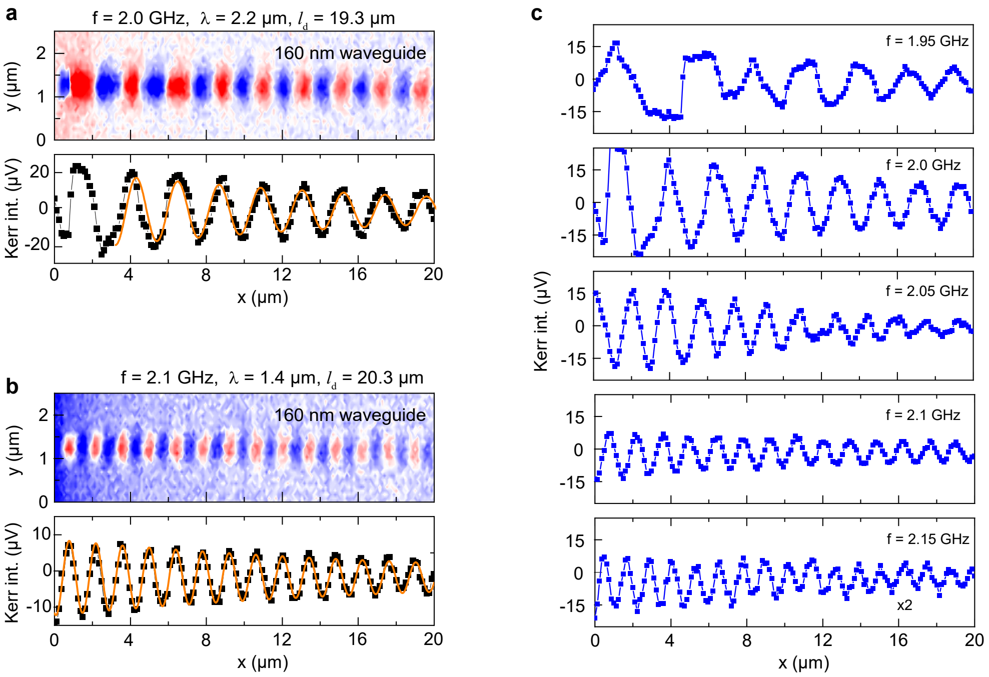

Figure S4. (a and b) SNS-MOKE microscopy maps and line profiles of propagating spin waves in a 160-nm-wide waveguide at 2.0 GHz and 2.1 GHz. The orange lines depict fits to the experimental data using $C\exp(-|x|/l_{\mathrm{d}})\sin(2\pi x/\lambda+\phi)$. The fitted wavelength (λ) and spin-wave decay length (l_{d}) are given above the spin-wave maps. (c) SNS-MOKE spin-wave profiles for a 160-nm-wide waveguide at different frequency. All data are recorded at -25 mT external magnetic field.

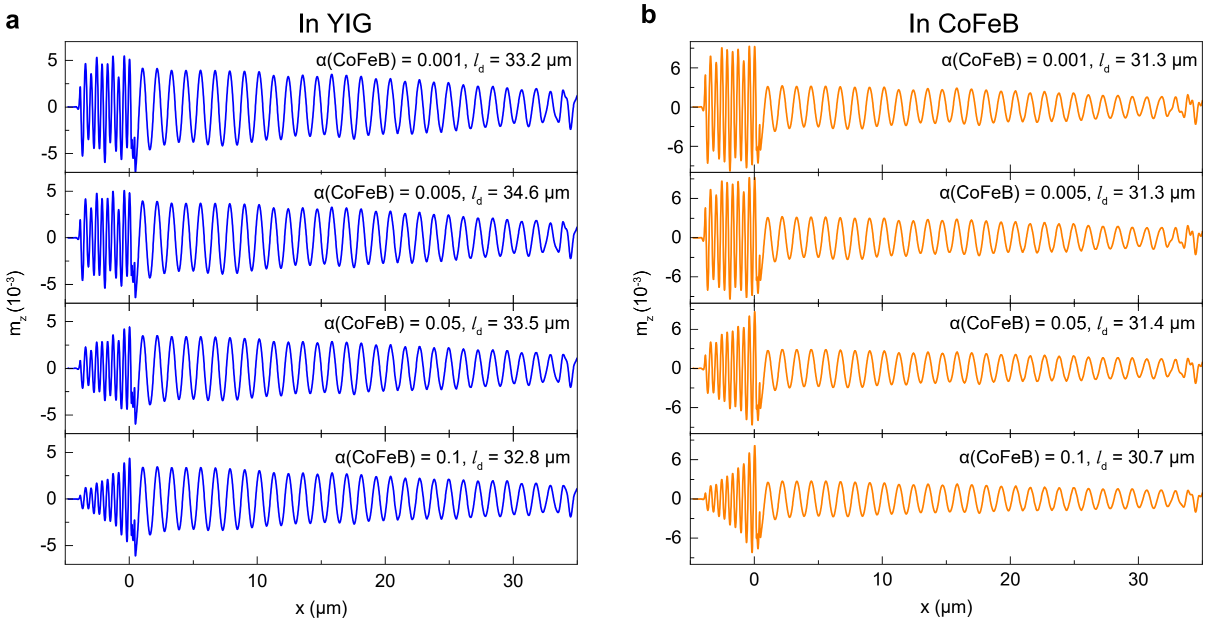

Figure S5. (a and b) Simulated line profiles of propagating spin waves in YIG and CoFeB for a 260-nm-wide waveguide at 1.84 GHz. In the simulations, the damping parameter of YIG is fixed at 0.001 and the damping parameter of CoFeB is varied from 0.001 to 0.1. The continuous YIG film is 66 nm thick and the CoFeB nanostripe is 24 nm thick. The spin-wave decay length does not vary much with the damping parameter of the CoFeB stripe.

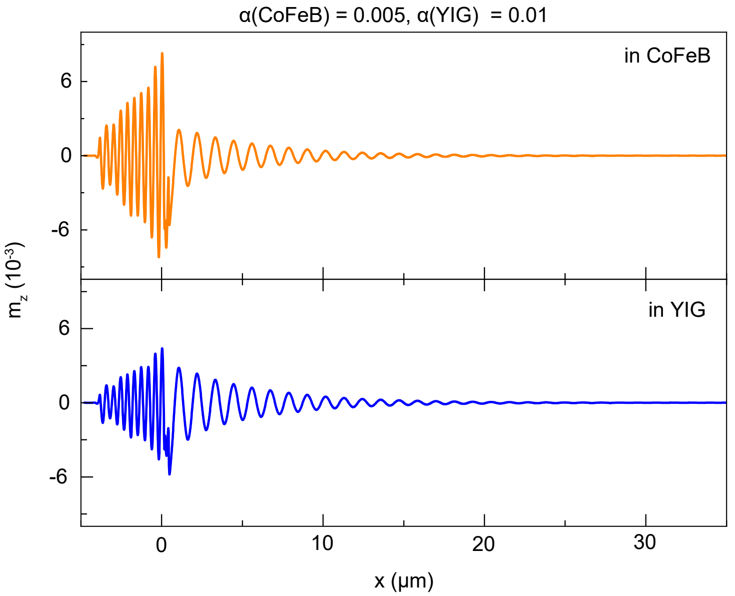

Figure S6. Simulated line profiles of propagating spin waves in YIG and CoFeB for the same waveguiding structure as in Figure S5. The damping parameter of YIG is enlarged to from 0.001 to 0.01. As a result the decay length decreases from 34.6 μm to 5 μm.

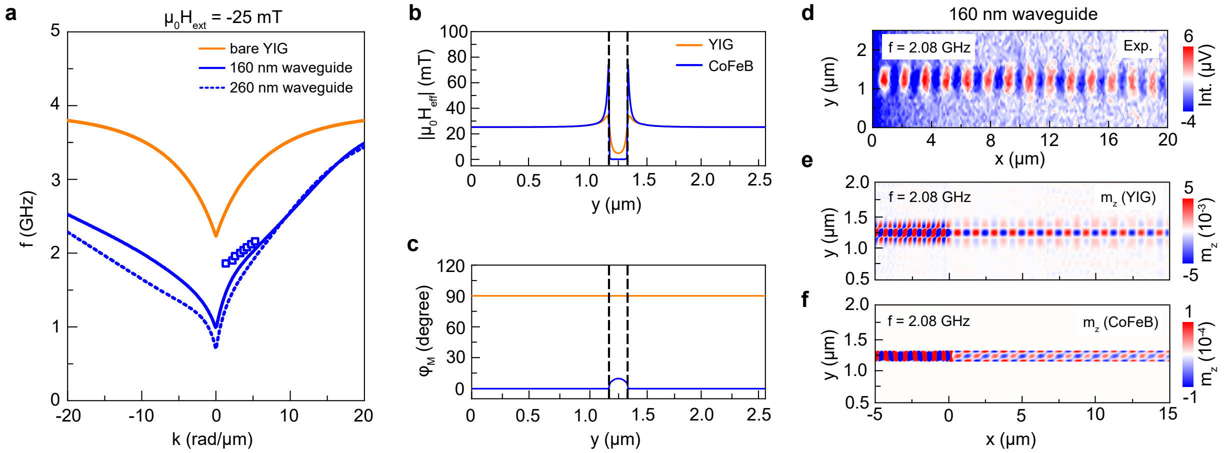

Figure S7. (a) Measured (empty symbols), calculated (solid lines), and simulated (solid squares) spin-wave dispersion relations for a 66-nm-thick bare YIG film and a 160-nm-wide waveguide. For comparison, the calculated spin-wave dispersion relation for a 260-nm-wide waveguide is also shown (dashed line). (b and c) Simulated effective field and magnetization angle in the YIG film and a 160-nm-wide CoFeB stripe. The magnetization angle is defined with respect to the x axis. The vertical dashed lines indicate the position of the CoFeB stripe. (d-f) Measured and simulated spin-wave maps for a 160-nm-wide waveguide at 2.08 GHz. The simulations in (e) and (f) depict the spin-wave mode in YIG and CoFeB, respectively. All data are obtained for a −25 mT magnetic field.

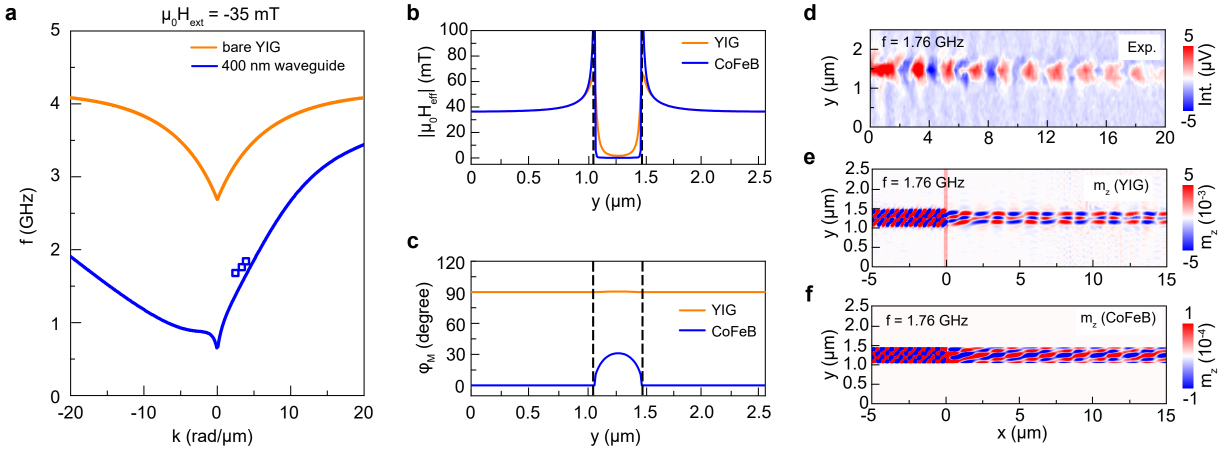

Figure S8. (a) Measured (empty symbols) and calculated (solid lines) spin-wave dispersion relations for a 66-nm-thick bare YIG film and a 400-nm-wide waveguide. (b and c) Simulated effective field and magnetization angle in the YIG film and a 400-nm-wide CoFeB stripe. The magnetization angle is defined with respect to the x axis. The vertical dashed lines indicate the position of the CoFeB stripe. (d-f) Measured and simulated spin-wave maps for a 400-nm-wide waveguide at 1.76 GHz. The simulations in (e) and (f) depict the spin-wave mode in YIG and CoFeB, respectively. All data are obtained for a −35 mT magnetic field.

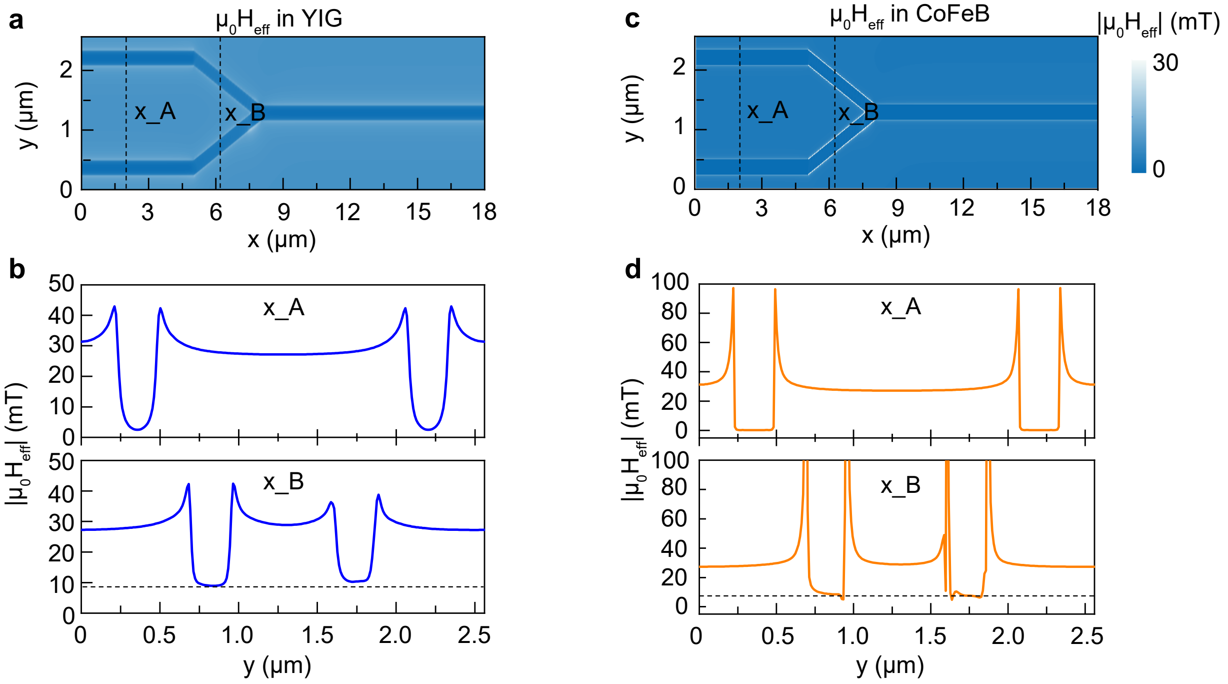

Figure S9. (a) Simulated spatial distribution of the effective magnetic field ($\mu_0 H_{eff}$) in a 260-nm-wide Y-shaped waveguide. The external magnetic field is -25 mT. The data are extracted for the top suface of the YIG film underneath the CoFeB stripe. (b) Corresponding line profiles of the effective magnetic field in the straight arms at position A ($x_A = 3$ μm) and in the tilted arms at position B ($x_B = 6.3$ μm). The effective magnetic field is the same in the two straight arms, but differs in the two tilted arms. (c and d) Same plots as in (a, b) but now for the effective magnetic field in the CoFeB stripe.

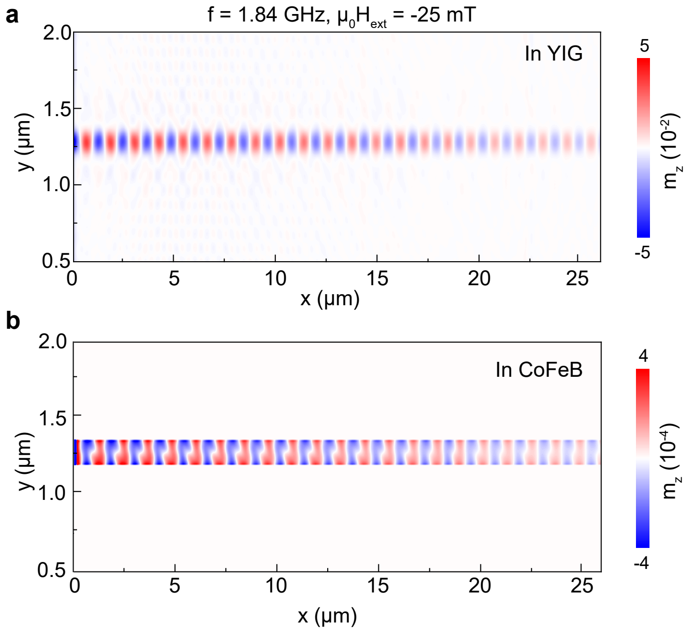

Figure S10. (a and b) Simulated spin-wave maps in YIG and CoFeB for a 160-nm-wide waveguide at 1.84 GHz. The external magnetic field is -25 mT. The thickness of the continuous YIG film is 20 nm and the thickness of the CoFeB stripe is 15 nm.

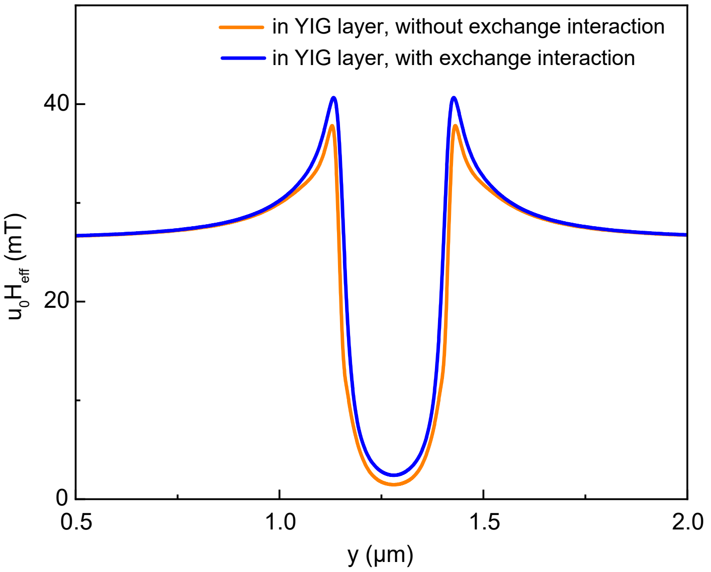

Figure S11. Simulated effective field in a 66-nm-thick YIG film with a 260-nm-wide CoFeB stripe on top. The CoFeB stripe is either seperated from the YIG film by a 6-nm-thick nonmagnetic spacer (orange curve) or in direct contact with the YIG film (blue curve). The external magnetic field is -25 mT.
